# Quality of Life and Factors Affecting It: A Study Among People Living Near a Solid Waste Management Facility

**DOI:** 10.3389/fpubh.2021.720006

**Published:** 2021-09-24

**Authors:** Long Thanh Phan, Gia Thanh Nguyen, Quynh Anh Dac Nguyen, Hieu Song Nguyen, Tin Trung Nguyen, Toru Watanabe

**Affiliations:** ^1^Melbourne School of Population and Global Health, The University of Melbourne, Melbourne, VIC, Australia; ^2^Faculty of Public Health, Hue University of Medicine and Pharmacy, Hue University, Hue, Vietnam; ^3^Faculty of Medicine, Duy Tan University, Da Nang, Vietnam; ^4^Department of General Administration, School of Medicine, Duy Tan University, Da Nang, Vietnam; ^5^Department of Food, Life and Environmental Sciences, Yamagata University, Yamagata, Japan

**Keywords:** quality of life, WHOQOL-BREF, environmental health, developing country, solid waste management facility

## Abstract

**Background:** The amount of waste generated has been increasing over the years. Meanwhile, the capacity of solid waste management facilities (SWMFs) for waste disposal does not meet the needs, resulting in adverse consequences on the natural environment and health of residents living near these plants, which can significantly degrade their quality of life (QoL). This study aims to evaluate the QoL of residents living near an SWMF and the potential impacts it has on the residents.

**Methods:** A cross-sectional descriptive study was conducted involving 801 subjects, aged 18 and above, who live near the SWMF of Hue City, Vietnam. The QoL of the subjects was quantitatively assessed using the WHO QoL assessment scale (WHOQOL-BREF). The general, health, and environmental factors influencing QoL were identified using bivariate and multivariate logistic regression analyses.

**Results:** About 22.6% of the subjects had a good QoL. In particular, the proportions for good psychological health (6.9%) and environment (13.6%) were low, indicating an influence of the SWMF. Significant factors that degraded the QoL of residents were less education defined by not graduating from high school (odds ratio, OR = 2.78; 95% CI = 1.09–7.06), poor health status (OR = 2.50; 95% CI: 1.56–4.01), dissatisfaction with water quality (OR = 2.41; 95% CI: 1.10–5.25), and unacceptance of the SWMF presence (OR = 1.70; 95% CI: 1.11–2.60). Moreover, subjects living within 2 km of the plant had dermatological diseases and digestive disorders more frequently than those who lived away from the plant. They also reported more complaints regarding water, air, and soil quality, which were likely due to the operation of the SWMF.

**Conclusions:** Burying and disposing of solid waste at the SWMF might lead to the degradation of the surrounding water and soil environments, and its collection and transportation are considered to cause odor and dust. The efforts of responsible authorities to strictly supervise and inspect these activities at the SWMF are essential, not only to protect the surrounding environment but also to improve the QoL of those who live nearby these plants.

## Introduction

The rapid breakthroughs of industry and technology have improved the quality of life (QoL) of people worldwide. However, as a result of these developments, waste management and treatment have become challenging for human life in the twenty-first century. It is estimated that the rate of waste accumulation is even faster than the rate of urbanization (1). For example, a study reported that humans have produced 8.3 billion tons of plastic waste since the beginning of the industry in the 1950's, but only a negligible 9% of them was recycled, 12% was burned, and the rest was discarded and buried worldwide (2). Such waste originates directly from daily activities and sometimes causes serious issues for the natural environment, e.g., air pollution, contaminated grounds, and results in poor human health, such as diarrhea, respiratory illnesses, or cancer (3–5). Furthermore, as demonstrated in research, the environment surrounding a solid waste management facility (SWMF) and the groundwater resource systems were also seriously damaged because of the long-term operation of such plants (6). Moreover, along with the unsustainable use of natural resources and inappropriate environmental management, the QoL of residents living near these SWMFs is also negatively affected due to their operation (6–9). Even in developed countries, despite the promulgation of policies and strategies on waste recycling and disposal, the effectiveness of these policies is very limited. For example, only 25.8% of the waste in the USA was recycled in 2017, and countries in the European Union were in a similar situation, with only 30% of the waste recycled every year (10–12).

The QoL of a population can be affected by environmental factors. For example, the QoL of people, particularly in terms of physical and psychological domains, was negatively impacted by air pollutants, toxins, noise, and dirtiness in a study in Colombia (13). In Vietnam, a typical developing country in Asia, the amount of domestic waste in urban areas nationwide was 38,000 tons per day in 2015, 85% of which was collected and treated (14). However, in the same year, the amount of domestic waste in rural areas was 32,000 tons per day, and only approximately 55% was collected (14). Domestic waste is mainly treated by burial (70%), leading to rising indignation among people living near unsanitary landfills (15). Furthermore, its detrimental effect on water and soil environments has not been investigated well. Moreover, burning without closed processing technology, which is likely to degrade the air environment, is another popular waste treatment (up to 28%) in Vietnam (16). These treatments of domestic waste have likely contributed to the ranking of the country of 77/132 countries in an overall environmental assessment by the Environmental Performance Index in 2015 (17). Specifically, air pollution (for which Vietnam is in 123rd place) has had the most detrimental effect (17). According to a study conducted in Ho Chi Minh City, Vietnam, in 2016, a 10 μg/m^3^ increment in air pollutants increased the risk of respiratory diseases from 0.7 to 8.0% and that of cardiovascular diseases from 0.5 to 4.0% (18).

Thua Thien Hue (T. T. Hue) Province is a center of economy and tourism in central Vietnam with a local population of over 1.1 million and more than 4 million tourists visiting in 2018 (19). The pressure for waste treatment in this province has invariably been high, and an SWMF started operating in 1999. The treatment capacity is approximately 480 tons per day and solves the problem of household waste disposal. In the treatment plant, the waste is sprayed with antiseptic chemicals, followed by a process of waste categorization, composting, and combustion. The inert waste is dumped into a nearby landfill. However, the increasing amount of waste has overloaded the capacity of the SWMF, which has likely caused environmental pollution with negative impacts on the QoL of people living in this area. Even in this situation, they seem to be unaware of its long-term impacts on their QoL due to a lack of knowledge of these issues or a belief that the authority is responsible for providing a better waste-recycling system (20).

While previous studies mainly assessed the impact of SWMFs on health problems experienced by nearby residents, other aspects such as mental health, social skills, and the environment have been scarcely discussed (21–25). Considering these aspects, this study aims to evaluate the QoL of residents as a comprehensive indicator of the impact of the SWMF for people living nearby. This study uses the WHO QoL assessment scale (WHOQOL-BREF), which has been used to measure the QoL, both for the general population and those suffering from different diseases (26–28). This study also aims to determine factors influencing the QoL of the residents. To the best of our knowledge, this is the first study to investigate the QoL of people living near an SWMF in the Southeast Asian region.

## Materials and Methods

### Study Design

This cross-sectional descriptive study was conducted from May to August 2019 in a town near Hue City, the capital of T. T. Hue Province in Vietnam. The required sample size was calculated as 768, based on the previous research (29). A multistage stratified sampling method was used to select participants. First, the town involved in this study, which comprises 12 wards, was separated into two regions based on the distance from the SWMF (one within 2 km and the other distance away from the SWMF). Then, two wards from each region were randomly selected, and 10 hamlets were randomly isolated from the four selected wards, and the number of subjects from each hamlet was determined to correspond to its population (Appendix 1). Only those subjects in compliance with the following criteria were included in the study (*n* = 801): (1) aged 18 or above, (2) had lived continuously in the target area for at least 6 months before the study, and (3) willing to be involved in the study. Those who were in a state of cognitive impairment, difficult to contact, suffering from a mental illness, hearing or speech impaired, and those who could not control their actions and thoughts mentally were excluded. This exclusion may have contributed to the overestimation of QoL by neglecting a certain number of people with low QoL, although its contribution cannot be considered.

### Measures and Instruments

The WHO QoL assessment scale (WHOQOL-BREF) was used (30). The QoL was quantified based on four main domains, namely, physical health, mental health, environment, and social relationships. This scale is one of the most widely used tools in QoL research as it enables us to assess individual perceptions in the context of their culture, personal goals, standards, and concerns (30, 31), and it has been widely field-tested and validated (32, 33). The WHOQOL-BREF includes 26 items from four aforementioned main domains with a relatively high consistency (Cronbach's alpha = 0.826). These facets were scored on a Likert scale of 1–5 with 1 = very poor, 2 = poor, 3 = neither poor nor good, 4 = good, and 5 = very good; and 1 = very satisfied, 2 = dissatisfied, 3 = neither dissatisfied nor satisfied, 4 = satisfied, and 5 = very satisfied; 1 = not at all, 2 = a little, 3 = a moderate amount, 4 = very much, and 5 = extremely; or 1 = never, 2 = seldom, 3 = quite often, 4 = very often, and 5 = always (30). Then, we used a specific formula to compute scores for each domain based on this Likert scale (Appendix 2). The overall evaluation of QoL was determined by averaging the scores of the four domains. The QoL was assessed based on the scores obtained, following a previous study of the QoL of Indian women, where the following criteria were applied: those who obtained scores <33.3, 33.3–66.7, and >66.7% were judged to have poor, average, and good QoL, respectively (34). In this study, the assessment was simplified by comparing the score <66.7% with that >66.7% to understand whether the subjects have a good QoL or not.

To determine factors influencing the QoL, general characteristics of the research subjects, including gender, age, marital status, economic status, occupation, educational level, number of persons living together, and duration of their living in the target area, were obtained through direct interviews. The subjects were also asked orally about their general health status, past illnesses, treatment history, and satisfaction levels with air, water, and soil qualities, and noise in the living environment. The interviewers, who were 5th-year students at Hue University of Medicine and Pharmacy, were well-trained to minimize potential biases in the answers collected from the subjects. In the training, the students received detailed explanations about the objectives of the research, the structure of the questionnaires, and how to avoid obstacles during the interview, followed by trial interviews with eight local people under our supervision to strengthen their skills. During the trial interviews, we evaluated their level of proficiency and understanding of the questionnaire and their interview skills. Then, only those who thoroughly understood the questionnaire had strong interviewing capabilities, and appropriate attitudes to local people were assigned as interviewers.

As another environmental factor, the residential distance from the SWMF was considered by categorizing the subjects into two groups, with Group 1 (*n* = 405) comprising those living ≤2 km from the SWMF, and Group 2 (*n* = 396) those living >2 km from the SWMF.

### Statistical Analysis

A chi-squared test was performed to analyze the associations between the score of the overall QoL evaluation and possible factors influencing it. A *P* < 0.05 was considered statistically significant. Subsequently, multivariate logistic regression (MLR) analysis was performed to evaluate the independent associations between the overall QoL and variables, which were significantly associated in the previous analysis. The odds ratio (OR) was used to assess the strength of the associations. SPSS 18.0 (developed by IBM Corporation, New York, USA) was used for all statistical analyses.

### Research Ethics

This study was approved by the Hue University of Medicine and Pharmacy and the local authorities in the area where the study was conducted. Written informed consent was obtained from all participants after clearly introducing the survey process. The research subjects participated voluntarily and could refuse to participate or withdraw from the interview at any time. The data collected were used for scientific purposes only, and all information related to the subjects was encrypted and kept confidential.

## Results

### QoL Assessment of the Research Subjects

Table 1 shows the QoL of the research subjects assessed on the WHOQOL-BREF scale. The overall assessment illustrates that only 22.6% of the residents had a good QoL. Physical health and social relationships contributed positively to the QoL, despite the fact that the mean scores were lower than the criterion (66.7). However, factors that led to lower QoL were clearly related to psychological health and environment, which may be attributed to the operation of the studied SWMF.

**Table 1:** Quality of life (QoL) of the research subjects on the WHOQOL-BREF scale (*n* = 801).

**Aspects**	**Mean score ± SD**	**Subjects with good QoL (%)**
Physical health	62.7 ± 12.4	41.8
Psychological health	58.0 ± 7.7	6.9
Social relationships	65.7 ± 13.6	45.2
Environment	56.8 ± 9.8	13.6
Overall evaluation	60.8 ± 7.7	22.6

### General Characteristics of the Research Subjects

Table 2 shows the general characteristics of the research subjects. The numbers of female and male subjects were approximately equal in this study. The average age of the subjects was 45.9 years, and one-fifth of them were over 60 years old at the time of this study. Furthermore, 88.6% of the subjects were married, and only 3.4% of them lived alone. Moreover, 4.1% lived in difficult economic circumstances, and more than 90% of the subjects had lived in the study area for more than 5 years.

**Table 2:** General characteristics of the research subjects (*n* = 801) and the association with overall QoL based on the chi-squared test.

**Factors**		**All**		**Overall QoL**		***P***
		***N***	**%**	**Not good (*n* = 620) (%)**	**Good (*n* = 181) (%)**	
Gender	Women	427	53.3	344 (53.4)	83 (45.9)	0.052
	Men	372	46.4	274 (46.4)	98 (54.1)	
	Others	2	0.2	2 (0.2)	0 (0.0)	
Age	<60	622	77.7	475 (76.6)	147 (81.2)	0.191
	≥60	179	22.3	145 (23.4)	34 (18.8)	
Marital status	Not married	91	11.4	63 (10.2)	28 (15.5)	**0.048**
	Married	710	88.6	557 (89.8)	153 (84.5)	
Living alone	No	774	96.6	597 (96.3)	177 (97.8)	0.325
	Yes	27	3.4	23 (3.7)	4 (2.2)	
Educational background	Unschooled	70	8.7	64 (10.3)	6 (3.3)	**<0.001**
	Primary school	155	19.4	135 (21.8)	20 (11.0)	
	Secondary school	217	27.1	170 (27.4)	47 (26.0)	
	High school	195	24.3	146 (23.5)	49 (27.1)	
	University/postgraduate	164	20.5	105 (16.9)	59 (32.6)	
Financial status	Poor	33	4.1	30 (4.8)	3 (1.7)	0.058
	Average or above	768	95.9	590 (95.2)	178 (98.3)	
Time living in the study area	Under 1 year	6	0.7	3 (0.5)	3 (1.7)	0.169
	1–5 years	65	8.1	52 (8.4)	13 (7.2)	
	5–10 years	68	8.5	48 (7.7)	20 (11.0)	
	Above 10 years	662	82.6	517 (83.4)	145 (80.1)	
Self-report health status	Not satisfied	351	56.2	312 (50.3)	39 (21.5)	**<0.001**
	Satisfied	450	43.8	308 (49.7)	142 (78.2)	

*Bold value means the P < 0.05*.

Table 2 also shows the associations between marital status, educational background, self-reported health status, and the overall QoL (*P* < 0.05). Being married, having a higher educational degree, and being in good health were identified as significant factors that determined a better QoL.

### Health Status of the Research Subjects

The aforementioned analysis revealed that 56.2% of the subjects were not satisfied with their current health status. Table 3 shows the health problems reported by the subjects, which might be the possible reasons for their dissatisfaction. Health problems with a high incidence reported in the study area included musculoskeletal diseases (27.1%), chronic diseases (25.3%), and digestive disorders (25.0%). Meanwhile, the subjects who reported a “not good” QoL had higher incidences of musculoskeletal, respiratory, and chronic diseases (*P* < 0.05) than the other subjects; however, no significant difference was found in terms of digestive disorders.

**Table 3:** Health issues of the research subjects (*n* = 801) and the association with QoL based on the chi-squared test.

**Diseases**	**All subjects (*****n*** **=** **801)**		**Overall QoL**				***P* value**
			**Not good (*****n*** **=** **620)**		**Good (*****n*** **=** **181)**		
	**Suffered *n* (%)**	**Non-suffered *n* (%)**	**Suffered *n* (%)**	**Non-suffered *n* (%)**	**Suffered *n* (%)**	**Non-suffered *n* (%)**	
Respiratory	145 (18.1)	656 (81.9)	127 (20.5)	493 (79.5)	18 (9.9)	163 (90.1)	**0.001**
Digestion	200 (25.0)	601 (75.0)	156 (25.2)	464 (74.8)	44 (24.3)	137 (75.7)	0.816
Dermatology	131 (16.4)	670 (83.6)	99 (16.0)	521 (84.0)	32 (17.7)	149 (82.3)	0.584
Chronic diseases	203 (25.3)	598 (74.7)	176 (28.4)	444 (71.6)	27 (14.9)	154 (85.1)	**<0.001**
Allergy	56 (7.0)	745 (93.0)	40 (6.5)	580 (93.5)	16 (8.8)	165 (91.2)	0.268
Blood	52 (6.5)	749 (93.5)	45 (7.3)	575 (92.7)	7 (3.9)	174 (96.1)	0.103
Musculoskeletal	217 (27.1)	584 (72.9)	187 (30.2)	433 (69.8)	30 (16.6)	151 (83.4)	**<0.001**

*Bold value means the P < 0.05*.

### Factors Influencing the QoL of the Research Subjects: Results of Multivariate Logistic Regression (MLR) Analysis

Table 4 shows the results of the MLR analysis conducted to identify factors that influence the overall QoL of the research subjects. This analysis involved only those factors that were significant in the chi-squared test (Tables 2, 3; Appendix 3). The results showed that the overall QoL was influenced by the educational background, with those graduating from high school (OR = 2.78; 95% CI: 1.09–7.06; *P* = 0.032) and university (OR = 3.89; 95% CI: 1.52–9.99; *P* = 0.005) having a significantly better QoL. Independent of the educational background, satisfaction with general health status significantly increased the QoL (OR = 2.50; 95% CI: 1.56–4.01; *P* < 0.001).

**Table 4:** Factors affecting the quality of life of the subjects (*n* = 801) as the result of multivariate logistic regression analysis.

**Factors**		**OR**	**95% CI**	***p*-value**
Educational background	Unschooled	1		
	Primary school	1.58	0.59–4.23	**0.366**
	Secondary school	2.44	0.96–6.19	**0.060**
	High school	2.78	1.09–7.06	**0.032**
	University/Post-graduate	3.89	1.52–9.99	**0.005**
Marital status	Not married	1		
	Married	1.05	0.62–1.77	0.871
Self-report health status	Not satisfied	1		
	Satisfied	2.50	1.56–4.01	**<0.001**
Respiratory diseases	Suffered	1		
	Non-suffered	1.51	0.86–2.64	0.152
Chronic diseases	Suffered	1		
	Non-suffered	0.97	0.57–1.65	0.912
Musculoskeletal diseases	Suffered	1		
	Non-suffered	1.22	0.76–1.97	0.414
Water quality	Not satisfied	1		
	Satisfied	2.41	1.10–5.25	**0.027**
Impact of the solid waste management facility	Not accepted	1		
	Accepted	1.70	1.11–2.60	**0.015**

*Bold value means the P < 0.05*.

Among the environmental factors, water quality had a significant relationship with QoL. The QoL of subjects who were unsatisfied with the water quality was significantly lower than that of subjects who expressed satisfaction (OR = 2.41; 95% CI: 1.10–5.25; *P* = 0.027). The existence of the SWMF was another relevant factor, as suggested by the fact that the subjects who reported being irritated by the plant had a lower QoL than those who reported “acceptance” for it (OR = 1.70, 95% CI: 1.11–2.60; *P* = 0.015).

## Discussion

Only a small proportion (22.6%) of the 801 participants was found to have a good QoL. The subjects in Group 1 reported poorer physical health than those in Group 2 (*P* = 0.001) (Appendix 4). A previous study in Korea also mentioned that a residence nearby a garbage-dumping site was negatively correlated with the physical domain of the QoL (35). Moreover, 57.8% of those in Group 1 thought that the operation of the plant had a negative impact on their lives (Appendix 5). This result is similar to a study in South Africa, in which 70 and 56% of residents living closer to the landfill site identified the deposition of municipal solid waste in landfills as a serious problem and had fears for their health in the future (36). In this study, although the QoL score for physical health was relatively high, significant differences in people suffering from dermatology diseases and digestive disorders were found between the two residential groups (*P* < 0.05) (Appendix 6). In contrast, the previous studies did not report any increases in illness (21) or demonstrate an increase in primarily respiratory diseases, eye irritation, and weakness of the body in populations living near wastewater treatment plants (23, 36). This could be attributed to different impacts of SWMFs and wastewater treatment plants, and local factors, such as the living environment, health status, climate, geography, genetics, and immune status of the population.

The QoL score of psychological health was relatively low (Table 1) regardless of the distance from the SWMF (Appendix 4). A higher QoL for this factor among senior residents was found in a previous study in Canada (24). It is possible that the participants with higher education might be able to lead a more comfortable life even under the impact of the SWMF. In addition, a previous study conducted in Greece noticed that the frequency of being in a bad mood, being angry, and getting sick reported by the subjects was significantly higher among residents living close to a wastewater treatment plant than all others (21). Although the impact of a Greek wastewater treatment plant differs from that of the SWMF studied here, the result from the Greek study, in which the distance from the wastewater treatment plant might affect greatly the QoL in terms of psychological health, should be also considered when referring to the impact of the SWMF in this study. The psychological QoL was not significantly affected by the distance because the subjects in Group 2 were also psychologically stressed by the SWMF or were there other factors for the low QoL commonly found in both groups? Nevertheless, as aforementioned, the number of people with good psychological QoL in the study area was negligible. However, psychological QoL should not be overlooked due to related impacts on residents who live near the SWMF, in which air pollutants may cause neuropathies such as memory disturbances, sleep disorders, anger, fatigue, head tremors, blurred vision, and slurred speech, as well as affecting the dopamine system, glutamate system, and N-methyl-D-Aspartate (37).

Regarding the environment, the satisfaction of residents with the quality of their living area was the second lowest (13.6%) in both Groups 1 and 2 (Table 1). A previous study in Korea also indicated the environment as being significantly associated with a lower QoL of people living near a garbage-dumping site (35). Water quality is one of the environmental factors related to the QoL. Most of the research subjects used tap water for domestic purposes; however, 13.4% used water from wells and ponds for animal breeding and cultivation, making them vulnerable to the possible impacts of the SWMF on water quality. The most common visible changes in water quality were strange odors (61.8%) and colors (55.9%) for Group 1 (Appendix 7). Many subjects reported that the water quality had changed markedly after the construction of the SWMF, especially in ponds and groundwater from drilled wells. Although this study did not analyze the water quality of the study site, many studies also indicated detrimental effects of the SWMF on the water quality by showing the inevitable presence of bacteria and heavy metals in water samples near the SWMF (23, 38, 39). The presence of chemicals, heavy metals, and pathogens in water was one of the key factors that determined the risks for human health, which was indicated by the significant differences in the dermatological diseases and digestive disorders between Groups 1 and 2 (Appendix 6). These results were somewhat similar to the results of a study in Pakistan, which identified water-borne diseases, including diarrhea, cholera, typhoid, paratyphoid, hepatitis A, dermatitis, enteric fever, and many more as threats to the health of nearby residents, especially children and the elderly (40).

In addition, 64.9% of Group 1 residents reported experiencing polluted air around the SWMF, especially in the early morning, late evening, and after rain or weather changes, whereas only 39.4% in Group 2 experienced the same as Group 1 (Appendix 8). A study in Malaysia revealed that roughly 83.7% of respondents living within a 2 km radius of the landfill experienced a bad smell, which affected the tranquility and QoL (41). Furthermore, 90.9 and 4.6% of subjects in Groups 1 and 2, respectively, considered that odors and unpleasant air were caused by the SWMF, due to a great amount of unprocessed waste and the progress of combustion (Appendix 9). The study period from May to August was in the dry season characterized by a high temperature over 39°C and relatively low humidity (around 60% in the daytime), according to the provided data of weather from the Web Portal T. T. Hue (42, 43). With the dry and hot wind from the South or South-West in the study periods, air pollutants and odor are likely to have affected residents on the leeward side of the SWMF more significantly (44), although it cannot be examined with our data. This issue was significantly considered when a previous study in the greater Athens showed that air pollutant concentration within 1.5 km from the landfill was significantly above the WHO reference lifetime exposure health criteria (45).

Refuse dumps release bioaerosols in the atmosphere that are associated with pathogens known for causing fatal diseases such as cholera and diarrhea (46). Although there was no significant difference regarding respiratory diseases between the two residential groups in this research, this type of disease contributed significantly to the difference between having good and not good QoL (Table 3). This type of disease requires attention because of its latent dangers. Waste transportation, which can cause dust, was also identified as a negative factor affecting air quality. Although 12.6% of the subjects in Group 1 mentioned waste transportation, none of the subjects in Group 2 were reported as being affected by it (Appendix 10). This also requires attention because of the serious problems that waste transfer has caused, especially dust, which was a serious concern for people living near landfills (36, 47–49). The SWMF also caused negative effects on soil quality, in which 17.5% of subjects in Group 1 were negatively affected by the degraded soil quality, in contrast to Group 2 with 11.1% (*P* < 0.05) (Appendix 8). The contamination of soil may occur as leachate produced by water or liquid wastes moving into, through and out of the landfill; migration into adjacent areas can affect the site characteristics and environmental health extensively (50). Irrespective of the annoyed respondents in Group 1 regarding water, air, and soil environments, there was no correlation between the two groups in terms of noise (Appendix 8). This result is somewhat consistent with other studies, which found that residents do not consider noise from the landfill operation, including blowing refuse and truck noise, as serious problems (48, 51).

The MLR analysis involving all the subjects (Table 4) revealed that the significant factors influencing a low overall QoL score were poor educational background, dissatisfaction with health status and water quality, shorter distance from the plant, and unacceptance of the plant. These factors were also significant in Group 1 (Appendix 11), whereas they were not significant in Group 2 (Appendix 12). In addition, in Group 1, higher-educated people had better QoL. The higher the level of education, the higher the perceived negative effects of the landfill operation, of the more education impacts their perception and independent solutions to the negative impact from the SWMF (52). The overall QoL was negatively affected by health status in both the groups, resulting in the lower QoL in terms of physical health in Group 1 (Appendix 4). Musculoskeletal diseases, which were significant factors regardless of the distance from the SWMF, were not associated with the SWMF when conducting the MLR analysis. Although there are no significant differences between musculoskeletal diseases along with respiratory and chronic diseases and QoL, an association between these diseases and the distance to the SWMF was found in previous studies (23, 36, 49). Therefore, substantial attention should be paid to the relationship between health issues and the distance to the SWMF. The dissatisfaction with water quality and unacceptance of the SWMF were not significant factors in Group 2, which indicated the extent of impacts of this plant. Meanwhile, these factors significantly affected the QoL of people in Group 1 (Appendix 11) in consistency with other studies (23, 48).

## Strengths and Limitations

This study comprehensively evaluated the possible impacts of the SWMF on the QoL of people living in the study area, such as human health and environmental issues. A strong point of this research is that it provides precise data on the QoL of people by applying the WHOQOL-BREF, which has been widely used in previous studies. In addition, dividing the study area into two different regions based on the distance from the SWMF enabled us to highlight the possible impacts and relevant factors of this plant.

On the other hand, we did not incorporate other facilities, such as a paper factory, fish sauce company, and plastic factory, which might impact the QoL of local people. In addition to the responses from local people, because T. T. Hue Province was determined to have poor waste management and collection, our priority was given to the SWMF (53). Furthermore, we excluded people who do not meet the relatively strict requirements for being interviewed, which, although there were few, may have limited the study.

There are a few more limitations to this study. The results based on the data, which were collected in a short time, may not represent the seasonal variations. For example, weather-related factors such as temperature, humidity, and wind direction could be affected by seasons, underlying the necessity of follow-up longitudinal studies to examine the seasonal difference in the impact of SWMF. Moreover, this cross-sectional study cannot detect the causal relationship between the impact of SWMF and QoL. The absence of air, water, and soil quality measurements in this study also limits the discussion regarding the impact of the SWMF on environmental and human health. Monitoring the contaminants in these environments from the SWMF requires further investigation for a more reliable health risk assessment.

## Conclusions

This study comprehensively assessed the impact of an SWMF in Hue City, Vietnam, on the QoL of its residents in terms of physical health, psychological health, social relationships, and the environment using the WHOQOL-BREF. The overall QoL was lower compared to the general criteria because of low scores in psychological health and environment. Among the possible influencing factors in these aspects, the residential distance from the SWMF was significant and shown to contribute to a lower QoL, along with poor education, dissatisfaction with health status and water quality, and unacceptance of the SWMF. Dermatological diseases and digestive disorders were reported more frequently, corresponding to the reported degradation of water quality, among those who live near the SWMF. Air pollution, such as odor and dust, and degraded soil quality also seem to make the plant unacceptable to residents.

Burying and disposing waste at the SWMF could degrade surrounding water and soil environments, and its collection and transportation are believed to cause odor and dust.

Based on the findings in this study, the efforts of responsible authorities to strictly supervise and inspect these activities at the SWMF can not only protect the surrounding environment but also improve the QoL of those who live near the plant. Their possible supervisions include updating the waste treatment technology, relocating the burial site containing unprocessed garbage and the combustion facility at least 5 km from residential areas (54), and prohibiting people from approaching the SWMF. In addition, other solutions to relieve the effects of the SWMF operation on the air quality of residential areas should be concerned, such as using panels to cover waste when it is transported to the SWMF to alleviate odors from waste trucks. Moreover, the authorities can also consider controlling dust emission from waste transportation by regularly spraying water on the soil (55).

## Data Availability Statement

The original contributions presented in the study are included in the article/Supplementary Material, further inquiries can be directed to the corresponding author/s.

## Ethics Statement

The studies involving human participants were reviewed and approved by Hue University of Medicine and Pharmacy, Vietnam. The patients/participants provided their written informed consent to participate in this study.

## Author Contributions

LP, GN, QN, HN, TN, and TW: conception and design. LP, QN, HN, and TN: collection and assembly of data. LP, GN, QN, HN, TN, and TW: data analysis and interpretation. LP, GN, and TW: writing, review, and editing. All authors read and approved the final manuscript.

## Funding

This work was supported by JSPS KAKENHI [Grant Number 19H01144], JSPS Core-to-Core Program, and SEI Group CSR Foundation.

## Conflict of Interest

The authors declare that the research was conducted in the absence of any commercial or financial relationships that could be construed as a potential conflict of interest.

## Publisher's Note

All claims expressed in this article are solely those of the authors and do not necessarily represent those of their affiliated organizations, or those of the publisher, the editors and the reviewers. Any product that may be evaluated in this article, or claim that may be made by its manufacturer, is not guaranteed or endorsed by the publisher.

## Abbreviations

SWMF: Solid waste management facility
QoL: Quality of life
WHO: World Health Organization
WHOQOL-BREF: World Health Organization Quality of Life
T.T. Hue: Thua Thien Hue Province
OR: Odds ratio
CI: Confidence intervals
SD: Standard deviation.
