# Supplementary material for: Quality of Life and Factors Affecting It: A Study Among People Living Near a Solid Waste Management Facility
Source: Front Public Health. 2021 Sep 24;9:720006. doi: 10.3389/fpubh.2021.720006 (PMC8634782; doi:10.3389/fpubh.2021.720006)
Supplement: Supplementary file 1 [file Table_1.docx]

**Quality of life and factors affecting it: A study among people living near a solid waste management facility**

Long Thanh Phan ^1^, Gia Thanh Nguyen ^2*^,Quynh Anh Dac Nguyen ^3^, Hieu Song Nguyen ^4^, Tin Trung Nguyen ^2^, Toru Watanabe^5^

**APPENDICES**

**APPENDIX 1: The distribution of the sample size**

| **Sample area** | | **Sample size** |
| --- | --- | --- |
| **Ward** | **Hamlet** |  |
| A | 1* | 103 |
|  | 2* | 103 |
|  | 3* | 101 |
| B | 4** | 65 |
|  | 5** | 67 |
|  | 6** | 26 |
| C | 7** | 58 |
|  | 8* | 98 |
| D | 9** | 100 |
|  | 10** | 80 |
| Total | 10 | 801 |

*Note: * Region 1 (within 2 km from the SWMF); ** Region 2 (further than 2 km)*

**APPENDIX 2: Calculation of quality of life score**

1) In each domain, the mean value (M) of scores (1 to 5) corresponding to answers to questions is calculated. Then, the domain mean score (D) is calculated by the following formula:

D = (M x 4 – 4) x (100/16).

2) The overall evaluation is made with the score determined by averaging the four domain mean scores.

3) Table S1 is the example of calculation of quality of life score for a subject.

Table S1. Calculation of quality of life score for Subject #51.

| **Subject #51** | **Score of answer to each question** | | | | |
| --- | --- | --- | --- | --- | --- |
|  | **1** | **2** | **3** | **4** | **5** |
| 1. **Physical health** |  |  |  |  |  |
| Q.1.1 |  |  | ✓ |  |  |
| Q.1.2 |  |  |  | ✓ |  |
| Q.1.3 |  |  | ✓ |  |  |
| Q.1.4 | ✓ |  |  |  |  |
| Q.1.5 |  |  |  | ✓ |  |
| Q.1.6 |  |  | ✓ |  |  |
| Q.1.7 |  |  | ✓ |  |  |
| Domain mean score | $(\frac{21}{7}\times4-4)\times(\frac{100}{16})=50.00$ | | | | |
| 1. **Psychological health** |  |  |  |  |  |
| Q.2.1 |  |  |  | ✓ |  |
| Q.2.2 |  |  |  | ✓ |  |
| Q.2.3 |  | ✓ |  |  |  |
| Q.2.4 |  |  |  | ✓ |  |
| Q.2.5 |  |  | ✓ |  |  |
| Q.2.6 |  |  |  | ✓ |  |
| Domain mean score | $(\frac{21}{6}\times4-4)\times(\frac{100}{16})=62.50$ | | | | |
| 1. **Social relationship** |  |  |  |  |  |
| Q.3.1 |  |  |  |  | ✓ |
| Q.3.2 |  |  |  |  | ✓ |
| Q.3.3 |  | ✓ |  |  |  |
| Domain mean score | $(\frac{12}{3}\times4-4)\times(\frac{100}{16})=75.00$ | | | | |
| 1. **Environment** |  |  |  |  |  |
| Q.4.1 | ✓ |  |  |  |  |
| Q.4.2 |  |  |  |  | ✓ |
| Q.4.3 |  |  | ✓ |  |  |
| Q.4.4 | ✓ |  |  |  |  |
| Q.4.5 |  | ✓ |  |  |  |
| Q.4.6 |  |  |  | ✓ |  |
| Q.4.7 |  |  | ✓ |  |  |
| Q.4.8 |  |  | ✓ |  |  |
| Domain mean score | $(\frac{22}{8}\times4-4)\times(\frac{100}{16})=43.75$ | | | | |
| **Overall evaluation** | $\frac{50.00 + 62.50+ 75.00 + 43.75}{4}=57.81$ | | | | |

**APPENDIX 3: Satisfaction with environmental factors and its influence on the QoL**

| Factors | Not good QoL (n=620) | | Good QoL (n=181) | | *P* value |
| --- | --- | --- | --- | --- | --- |
|  | Unsatisfied (%) | Satisfied (%) | Unsatisfied (%) | Satisfied (%) |  |
| Air quality | 331 (53.4) | 289 (46.6) | 88 (48.6) | 93 (51.4) | 0.258 |
| Water quality | 84 (13.5) | 536 (86.5) | 8 (4.4) | 173 (95.6) | **0.001** |
| Soil quality | 95 (15.3) | 525 (84.7) | 20 (11.0) | 161 (89.0) | 0.149 |
| Noise | 397 (64.0) | 223 (36.0) | 118 (65.2) | 63 (34.8) | 0.774 |

**APPENDIX 4: QoL (mean** ± **SD) on WHOQOL-BREF scale of the research subjects in two groups characterized by distance from the SWMF**

| Aspects | Group 1 (n=405) | Group 2 (n=396) | Total (n=801) | *P**value |
| --- | --- | --- | --- | --- |
| Physical health | 61.0 ± 13.3 | 64.4 ±11.0 | 62.7 ± 12.4 | 0.01 |
| Psychological health | 58.2 ± 8.5 | 57.8 ± 6.8 | 58.0 ± 7.7 | 0.491 |
| Social relationships | 65.1 ± 14.3 | 66.3 ± 12.7 | 65.7 ± 13.6 | 0.751 |
| Environment | 56.5 ± 10.7 | 57.1 ± 8.7 | 56.8 ± 9.8 | 0.950 |
| Overall evaluation | 60.2 ± 8.7 | 61.4 ± 6.6 | 60.8 ± 7.7 | 0.087 |

*Group 1 (Subjects living within 2km from the SWMF) and Group 2 (the others)*

** Mann-Whitney U Test*

**APPENDIX 5: Respondents' perception of SWMF correlation to their QoL**

|  | Group 1 (n=405) | | Group 2 (n=396) | | *P* value |
| --- | --- | --- | --- | --- | --- |
|  | Negative  n (%) | Positive/No impact  n (%) | Negative  n (%) | Positive/No impact  n (%) |  |
| Impact of SWMF | 234 (57.8) | 171 (42.2) | 23 (5.8) | 373 (93.2) | **<0.001** |

*Group 1 (Subjects living within 2km from the SWMF) and Group 2 (the others)*

**APPENDIX 6: Diseases reported by the subjects in two groups characterized by distance from the SWMF**

| Diseases | Group 1 (n=405) | | Group 2 (n=396) | | *P* value |
| --- | --- | --- | --- | --- | --- |
|  | Suffered  n (%) | Non-  suffered  n (%) | Suffered  n (%) | Non-  suffered  n (%) |  |
| Respiratory | 79 (19.5) | 326 (80.5) | 66 (16.7) | 330 (83.3) | 0.297 |
| Digestion | 117 (28.9) | 288 (71.1) | 83 (21) | 313 (79) | **0.01** |
| Dermatology | 90 (22.2) | 315 (77.8) | 41 (10.4) | 355 (89.6) | **<0.001** |
| Chronic  diseases | 106 (26.2) | 299 (73.8) | 97 (24.5) | 299 (75.5) | 0.585 |
| Allergy | 32 (7.9) | 373 (92.1) | 24 (6.1) | 372 (93.9) | 0.307 |
| Blood | 33 (8.1) | 372 (91.9) | 19 (4.8) | 377 (95.2) | 0.054 |
| Musculoskeletal | 104 (25.7) | 301  (74.3) | 113 (28.5) | 283  (71.5) | 0.363 |

*Group 1 (Subjects living within 2km from the SWMF) and Group 2 (the others)*

**APPENDIX 7: Claims about the water supply reported by the subjects in two groups characterized by distance from the SWMF**

| Factors | Group 1 (n=68) | | Group 2 (n=24) | |
| --- | --- | --- | --- | --- |
|  | Disagree (%) | Agree (%) | Disagree (%) | Agree (%) |
| Lack of water | 10 (14.7) | 58 (85.3) | 2 (8.36) | 22 (91.7) |
| Strange odors | 42 (61.8) | 26 (38.2) | 16 (66.7) | 8 (33.3) |
| Strange colors | 38 (55.9) | 30 (44.1) | 4 (16.7) | 20 (83.3) |
| Impurities | 18 (26.5) | 50 (73.5) | 0 (0.0) | 24 (100.0) |
| Alum | 5 (7.4) | 63 (92.6) | 0 (0.0) | 24 (100.0) |
| Others | 3 (4.4) | 65 (95.6) | 4 (16.7) | 20 (83.3) |

*Group 1 (Subjects living within 2km from the SWMF) and Group 2 (the others)*

| Factors | Group 1 (n=405) | | Group 2 (n=396) | | *P* value |
| --- | --- | --- | --- | --- | --- |
|  | Non-satisfied (%) | Satisfied (%) | Non-satisfied (%) | Satisfied (%) |  |
| Air quality | 263 (64.9) | 142 (35.1) | 156 (39.4) | 240 (60.6) | **<0.001** |
| Water quality | 68 (16.8) | 337 (83.2) | 24 (6.1) | 372 (93.9) | **<0.001** |
| Soil quality | 71 (17.5) | 334 (82.5) | 44 (11.1) | 352 (88.9) | **0.01** |
| Noise | 263 (64.9) | 142 (35.1) | 252 (63.6) | 144 (36.4) | 0.701 |

**APPENDIX 8: Satisfaction with environmental factors in two groups characterized by distance from the SWMF**

*Group 1 (Subjects living within 2km from the SWMF) and Group 2 (the others)*

**APPENDIX 9: Sources of the odor issue reported by the subjects in two groups characterized by distance from the SWMF**

| Odor sources | Group 1 (n=198) | | Group 2 (n=66) | |
| --- | --- | --- | --- | --- |
|  | Agree (%) | Not agree (%) | Agree (%) | Not agree (%) |
| SWMF | 179 (90.4) | 19 (9.6) | 8 (12.1) | 58 (87.9) |
| Waste transportation | 19 (9.6) | 179 (90.4) | 10 (15.2) | 56 (84.8) |
| Others | 23 (11.6) | 175 (88.4) | 48 (72.7) | 18 (27.3) |

*Group 1 (Subjects living within 2km from the SWMF) and Group 2 (the others)*

**APPENDIX 10: Sources of the dust issue reported by the subjects in two groups characterized by distance from the SWMF**

| Dust sources | Group 1 (n=87) | | Group 2 (n=95) | |
| --- | --- | --- | --- | --- |
|  | Agree (%) | Not agree (%) | Agree (%) | Not agree (%) |
| Vehicles | 81 (93.1) | 6 (6.9) | 80 (84.2) | 15 (15.8) |
| Waste transportation | 11 (12.6) | 76 (87.4) | 0 (0.0) | 95 (100.0) |
| Others | 10 (11.5) | 77 (88.5) | 16 (16.8) | 77 (83.2) |

*Group 1 (Subjects living within 2km from the SWMF) and Group 2 (the others)*

**APPENDIX 11: Factors affecting the quality of life of people living in Group 1 (n = 405) as the result of multivariate logistic regression analysis**

| **Factors** | | **OR** | **95% CI** | ***P* value** |
| --- | --- | --- | --- | --- |
| **Educational background** | Unschooled  Primary school  Secondary  school  High school  University/  Post-graduate | 1  1.46  2.61  5.45  8.97 | 0.27-7.81  0.53-12.88  1.10-27.11  1.77-45.51 | 0.662  0.238  **0.038**  **0.008** |
| **Marital status** | Not married  Married | 1  0.90 | 0.44-1.83 | 0.763 |
| **Self-report health status** | Not satisfied  Satisfied | 1  2.90 | 1.36-6.13 | **0.005** |
| **Respiratory**  **diseases** | Suffered  Non-suffered | 1  1.56 | 0.72-3.38 | 0.260 |
| **Chronic diseases** | Suffered  Non-suffered | 1  0.97 | 0.43-2.18 | 0.937 |
| **Musculoskeletal diseases** | Suffered  Non-suffered | 1  0.41 | 0.20-0.85 | **0.016** |
| **Water quality** | Not satisfied  Satisfied | 1  5.49 | 1.60-18.90 | **0.007** |
| **Impact of the solid waste management facility** | Not accepted  Accepted | 1  2.62 | 1.52-4.49 | **<0.001** |

**APPENDIX 12: Factors affecting the quality of life of people living in Group 2 (n = 396) as the result of multivariate logistic regression analysis**

| **Factors** | | **OR** | **95% CI** | ***P* value** |
| --- | --- | --- | --- | --- |
| **Educational background** | Unschooled  Primary school  Secondary  school  High school  University/  Post-graduate | 1  1.84  2.75  1.60  2.58 | 0.52-6.45  0.84-8.96  0.48-5.30  0.78-8.57 | 0.343  0.094  0.441  0.122 |
| **Marital status** | Not married  Married | 1  1.71 | 0.67-4.40 | 0.262 |
| **Self-report health status** | Not satisfied  Satisfied | 1  2.48 | 1.24-4.93 | **0.010** |
| **Respiratory**  **diseases** | Suffered  Non-suffered | 1  1.56 | 0.64-3.81 | 0.328 |
| **Chronic diseases** | Suffered  Non-suffered | 1  0.92 | 0.43-2.00 | 0.838 |
| **Musculoskeletal diseases** | Suffered  Non-suffered | 1  4.74 | 1.98-11.35 | **<0.001** |
| **Water quality** | Not satisfied  Satisfied | 1  0.53 | 0.17-1.68 | 0.280 |
| **Impact of the solid waste management facility** | Not accepted  Accepted | 1  0.99 | 0.26-3.84 | 0.990 |
